# Brugada Phenocopy in a Critical Obstetric Patient: A Case Report

**DOI:** 10.1155/carm/9972483

**Published:** 2025-06-16

**Authors:** Manuel Enrique Rodríguez García, Yassel Arias Otamendy, Annia de la Caridad Aguirre Ruiz

**Affiliations:** ^1^Dr. Agostinho Neto Teaching General Hospital of Guantánamo, Guantánamo, Cuba; ^2^University of Medical Sciences of Guantánamo, Guantánamo, Cuba; ^3^Cuban Society of Anesthesiology and Resuscitation, Havana, Cuba; ^4^Latin American Confederation of Societies of Anesthesiology (CLASA), Lima, Peru; ^5^Cuban Society of Intensive Medicine and Emergencies, Havana, Cuba; ^6^Cuban Society of Cardiology, Havana, Cuba

**Keywords:** Brugada phenocopy, case report, critical obstetric patient, hyperkalemia, metabolic acidosis

## Abstract

Brugada phenocopy (BrP) is an electrocardiographic (ECG) alteration that mimics Brugada syndrome (BrS) but lacks the associated genetic predisposition. It manifests as a transient pattern induced by reversible factors such as electrolyte imbalances, internal environment disturbances, and the use of certain drugs. In critically ill patients, hyperkalemia and acidosis are common triggers of this phenomenon, affecting ventricular repolarization and generating an ECG pattern identical to BrS. This report describes the case of a 30-year-old female patient who, following a complicated cesarean section with hemorrhagic shock, developed BrP induced by hyperkalemia and metabolic acidosis. The patient initially exhibited a type 1 Brugada ECG pattern, which evolved into a type 2 pattern and ultimately normalized after correction of the underlying disorders through hemodialysis. This case highlights the importance of an accurate differential diagnosis, as misidentifying BrP as true BrS could lead to inappropriate interventions, such as the implantation of defibrillators.

## 1. Introduction

Brugada phenocopy (BrP) is a variant in which electrocardiographic (ECG) patterns identical to those of Brugada syndrome (BrS) are observed, without an associated genetic predisposition to the disease. First described in 2012, it is an ECG alteration induced by reversible external factors such as certain medications, electrolyte imbalances, and acid–base disturbances [1].

BrP induced by electrolyte and acid–base imbalances represents a clinically relevant condition, as these disturbances are common in critically ill patients, particularly hyperkalemia and metabolic acidosis, which occur more frequently in individuals with associated renal insufficiency [2–4].

It is essential to differentiate BrP, which results from the direct impact of hyperkalemia and acidosis on ventricular repolarization and is reversible upon correction of potassium levels and acid–base imbalance, from true BrS [5]. Misidentification of genuine BrS could lead to inappropriate interventions, such as defibrillator implantation or the use of antiarrhythmic drugs [6].

This report presents the case of a critically ill obstetric patient who developed BrP secondary to hyperkalemia and metabolic acidosis. It highlights the importance of an accurate differential diagnosis and timely management in complex clinical settings.

## 2. Case Presentation

A 30-year-old female patient from a rural area, with no relevant personal or family medical history, was admitted to the hospital with a diagnosis of pregnancy at 40.3 weeks of gestation in the active phase of labor. Due to the risk of fetal distress, an urgent cesarean section was performed.

Following fetal extraction, the patient experienced profuse hemorrhage associated with uterine atony, necessitating an obstetric subtotal abdominal hysterectomy, followed by hypogastric artery ligation and damage control maneuvers, including abdominal cavity packing and the placement of two abdominal drains to manage residual bleeding.

**Figure 1:**
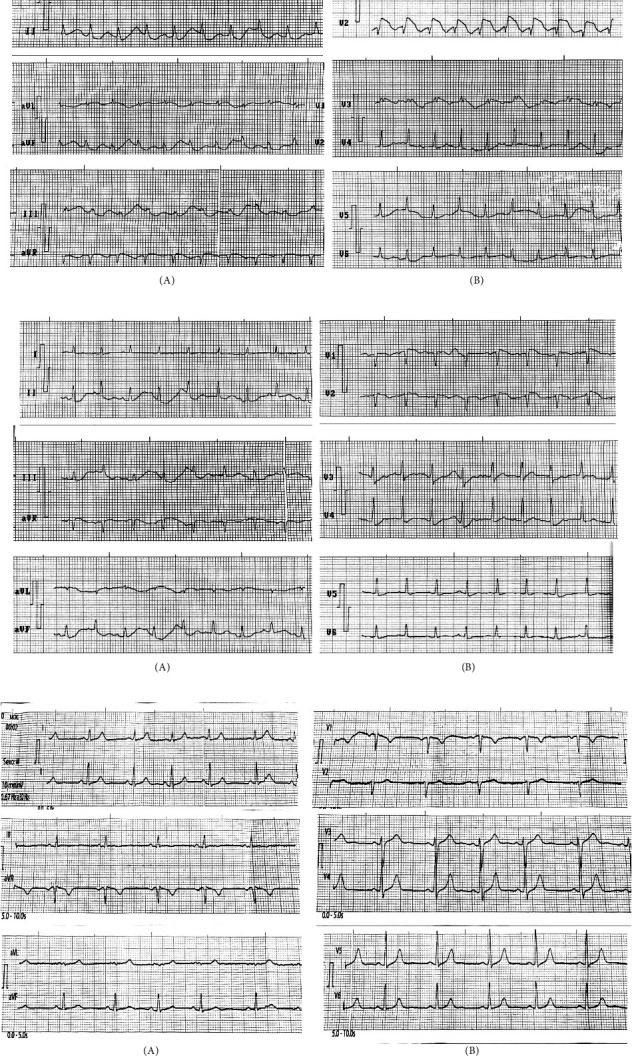
ECG with Brugada phenocopy. (a) Type 1 Brugada pattern. Ph: 7.11. K: 6.5 mmol/L. (b) ECG 2. Brugada pattern type 2. Ph: 7.29. K: 5.7. (c) ECG 3. Basal. K: 4.2. Ph: 7.4. (A): 12-lead ECG showing sinus tachycardia (150 bpm) with > 2 mm ST-segment elevation, coved type in V1 and V2 with inverted T waves (consistent with a type 1 Brugada pattern). (B): Electrocardiographic sequence of leads V1 and V2. The correction of acidosis and hyperkalemia was accompanied by a progressive normalization of the ECG, with resolution from a type 1 Brugada pattern to a type 2 pattern, and ultimately to a normal ECG.

During surgery, blood loss exceeded 50% of the total blood volume, requiring fluid resuscitation with crystalloids, colloids, fresh frozen plasma, and packed red blood cells. Despite these interventions, the severity of the hemorrhage led to signs of hemorrhagic shock with decreased urine output, initially consistent with prerenal acute kidney injury, which progressed to acute tubular necrosis with urgent dialysis criteria. Arterial blood gas and electrolyte analysis revealed progressive metabolic acidosis with an elevated anion gap due to hyperlactatemia, along with hyperkalemia, leading to BrP (Figure 1). The patient exhibited a type 1 pattern, characterized by an ST-segment elevation > 2 mm with a coved-type morphology in leads V1 and V2 and inverted T waves, identical to congenital BrS. The pattern evolved into type 2 and ultimately normalized after dialysis treatment and correction of acidosis and hyperkalemia. Subsequently, the electrocardiogram remained unchanged, with no evidence of recurrence of the type 1 pattern, and a complete resolution of the phenomenon induced by the underlying metabolic disturbances. Echocardiographic evaluation showed no global or segmental contractility abnormalities suggestive of myocardial ischemia.

Laboratory tests:
  • Hemoglobin: 81 g/L
  • Hematocrit: 0.27 L/L
  • Total bilirubin: 37.4 mmol/L
  • Direct bilirubin: 1.8 mmol/L
  • ALT (TGP): 2700 U/L
  • AST (TGO): 5051 U/L
  • Creatinine: 978 mmol/L
Coagulation profile:
  • Bleeding time: > 10 min
  • Clotting time: 25 min
  • Clot retraction: nonretractile
  • Platelets: 52 × 10 ^ 9/L
  • Fibrinogen: > 135 mg%
  • Activated partial thromboplastin time with kaolin: 85 s
  • Prothrombin time: control: 14 s and patient: 35 s

## 3. Discussion

BrP has been documented as a transient ECG alteration induced by electrolyte imbalances, drugs, and acidosis, which can lead to sudden cardiac death without the genetic predisposition characteristic of BrS [7, 8].

Hyperkalemia has been recognized as a triggering factor in some cases of BrP, especially when associated with acidosis. This combination can be linked to severe hemodynamic changes [5], which may worsen the prognosis of these patients in the context of hypovolemic shock with acute renal failure, as presented in this case.

Hyperkalemia can induce depolarization of the resting membrane potential, reducing the availability and proper function of sodium channels. The nonhomogeneous distribution of sodium currents, with a higher concentration in the right ventricular outflow tract, is associated with the loss of the action potential dome and the appearance of the Brugada pattern [9]. This phenomenon has been associated with a 40% increase in the incidence of malignant arrhythmias and an increase in mortality [10–12]. Furthermore, conditions such as acidosis may worsen this ECG pattern by altering the ionic balance and impairing sodium channel function, thereby exacerbating the electrical dysfunction of the heart and leading to ventricular dysfunction [10, 13, 14].

The ECG pattern of BrP in the context of hyperkalemia and metabolic acidosis is more common in critically ill patients, where these metabolic disturbances are frequent [15]. Previous studies have shown that potassium levels ≥ 6.5 mmol/L are a common cause of BrP [2]. In this case, a direct relationship between the severity of hyperkalemia and the expression of BrP was observed. As the severity of the potassium disturbance changed, the electrocardiogram transitioned from a type 1 Brugada pattern to type 2 and subsequently normalized as potassium levels returned to the normal range, suggesting a transient dysfunction of the sodium channel acquired through hyperkalemia.

It is important to highlight that the diagnostic criteria for BrP include not only the presence of a type 1 or type 2 Brugada ECG pattern and the identification of an underlying condition that normalizes the ECG pattern upon resolution, as evidenced in this case, but also a low clinical probability of true BrS. This low clinical probability is determined by the absence of symptoms, personal, and family medical history, as demonstrated in this case, where the provocation test with ajmaline was not considered necessary due to the patient's evolution and overall clinical context [16–19]. In patients with a low probability of true BrS, as evidenced in this case, the rate of positive results is very low [20]. In addition, it is important to note that this patient showed no contractility disorders on echocardiography that would suggest myocardial ischemia, reinforcing the notion that not all ST-segment elevations are indicative of myocardial infarction [21].

The recognition of BrP is crucial for providing timely treatment and establishing an accurate prognosis, as the correction of electrolyte and acid–base disturbances allows for complete resolution of the Brugada pattern. Therefore, the use of urgent dialysis constitutes a valuable therapeutic tool in this clinical context [22, 23].

## 4. Conclusions

This case highlights the importance of recognizing BrP induced by electrolyte and acid–base imbalances, particularly in critically ill patients experiencing severe metabolic disturbances such as hyperkalemia and metabolic acidosis. The rapid resolution of the ECG pattern after the correction of underlying disorders emphasizes that BrP is a reversible condition, unlike true BrS. This case underscores the need for an appropriate differential diagnosis to avoid inappropriate interventions, such as the use of defibrillators, in patients presenting ECG findings identical to BrS. Early identification and proper management of metabolic imbalances are essential to improving the prognosis of these patients by reducing the incidence of malignant arrhythmias, myocardial dysfunction, and death.

## Data Availability

The data that support the findings of this study are available on request from the corresponding author. The data are not publicly available due to privacy or ethical restrictions.

## References

[1] Baranchuk A., Nguyen T., Ryu M. H. (2012). Brugada Phenocopy: New Terminology and Proposed Classification. *Annals of Noninvasive Electrocardiology*.

[2] Xu G., Gottschalk B., Anselm D. (2018). Relation of the Brugada Phenocopy to Hyperkalemia (From the International Registry on Brugada Phenocopy). *The American Journal of Cardiology*.

[3] Doty B., Kim E., Phelps J., Akpunonu P. (2020). Pathophysiology of Hyperkalemia Presenting as Brugada Pattern on Electrocardiogram (ECG). *American Journal of Case Reports*.

[4] Seger S. (2021). Electrocardiogram (ECG) Changes of a Patient With Severe Hyperkalemia. *PAMJ Clinical Medicine*.

[5] Manne J., Garg J. (2021). Hyperkalemia Induced Brugada Phenocopy. *Journal of Arrhythmia*.

[6] Andorin A., Behr E., Denjoy I. (2016). Impact of Clinical and Genetic Findings on the Management of Young Patients With Brugada Syndrome. *Heart Rhythm*.

[7] Yılmaz E., Özdemir F. (2023). Brugada Phenocopy Induced by Hypovolemic Hyponatremia. *Cureus*.

[8] Hunuk A., Hunuk B., Kusken O., Onur O. (2016). Brugada Phenocopy Induced by Electrolyte Disorder: A Transient Electrocardiographic Sign. *Annals of Noninvasive Electrocardiology*.

[9] Zhang Z., Tranquillo J., Neplioueva V., Bursac N., Grant A. (2007). Sodium Channel Kinetic Changes that Produce Brugada Syndrome or Progressive Cardiac Conduction System Disease. *American Journal of Physiology—Heart and Circulatory Physiology*.

[10] Heckle M., Alsafwah S., Agarwal M. (2018). Multifactorial Brugada Phenocopy—Reply. *JAMA Internal Medicine*.

[11] Rivera-Juárez A., Hernández-Romero I., Puertas C. (2019). Clinical Characteristics and Underlying Electrophysiological Mechanisms of Brugada ECG in Patients With Severe Hyperkalemia. *American Heart Association Journals*.

[12] De Oliveira Neto N., De Oliveira W., Mastrocola F., Sacilotto L. (2019). Brugada Phenocopy: Mechanisms, Diagnosis, and Implications. *Journal of Electrocardiology*.

[13] Dahal K., Shrestha D., Hada R., Baral A., Sherpa K. (2020). Hyperkalemia Mimicking Brugada Pattern in Electrocardiogram: A Rare Case Report From Nepal. *Saudi Journal of Kidney Diseases and Transplantation*.

[14] Asatryan B. (2019). Cardiac Sodium Channel Dysfunction and Dilated Cardiomyopathy: A Contemporary Reappraisal of Pathophysiological Concepts. *Journal of Clinical Medicine*.

[15] Greffie E., Alhalaseh S., Zaremski L. (2023). Not All ST Elevation Is STEMI: Brugada Phenocopy Induced by Hyperkalemia. *Cureus*.

[16] Anselm D., Evans J., Baranchuk A. (2014). Brugada Phenocopy: A New Electrocardiogram Phenomenon. *World Journal of Cardiology*.

[17] Dendramis G. (2016). Brugada Syndrome and Brugada Phenocopy. The Importance of a Differential Diagnosis. *International Journal of Cardiology*.

[18] Gottschalk B., Baranchuk A. (2018). Brugada Phenocopy: Definition, Diagnosis, and Differentiation From True Brugada Syndrome. *Brugada Phenocopy: The Art of Recognizing the Brugada ECG Pattern*.

[19] Anselm D. D., Gottschalk B. H., Baranchuk A. (2014). Brugada Phenocopies: Consideration of Morphologic Criteria and Early Findings From an International Registry. *Canadian Journal of Cardiology*.

[20] Rolf S., Bruns H., Wichter T. (2003). The Ajmaline Challenge in Brugada Syndrome: Diagnostic Impact, Safety, and Recommended Protocol. *European Heart Journal*.

[21] Dendramis G., Petrina S., Baranchuk A. (2017). Not All ST-Segment Elevations Are Myocardial Infarction: Hyperkalemia and Brugada Phenocopy. *The American Journal of Emergency Medicine*.

[22] Monterrubio-Villar J., Llinares-Moya D. (2020). Brugada Phenocopy Induced by a Lethal Methanol Intoxication. *European Journal of Case Reports in Internal Medicine*.

[23] Hendsun H., Firmansyah Y., Setiawan I. (2022). Brugada Phenocopy that Lurks After Hemodialysis: A Case Report. *Indian Journal of Clinical Cardiology*.

